# Stable and destabilized GFP reporters to monitor calcineurin activity in *Saccharomyces cerevisiae*

**DOI:** 10.15698/mic2020.04.713

**Published:** 2020-02-05

**Authors:** Jutta Diessl, Arpita Nandy, Christina Schug, Lukas Habernig, Sabrina Büttner

**Affiliations:** 1 Department of Molecular Biosciences, the Wenner-Gren Institute, Stockholm University, SE-10691 Stockholm, Sweden.; 2 Institute of Molecular Biosciences, University of Graz, A-8010 Graz, Austria.

**Keywords:** calcineurin, calcium signaling, yeast, destabilized GFP, Crz1, reporter, flow cytometry

## Abstract

The protein phosphatase calcineurin is activated in response to rising intracellular Ca^2+^ levels and impacts fundamental cellular processes in organisms ranging from yeast to humans. In fungi, calcineurin orchestrates cellular adaptation to diverse environmental challenges and is essential for virulence of pathogenic species. To enable rapid and large-scale assessment of calcineurin activity in living, unperturbed yeast cells, we have generated stable and destabilized GFP transcriptional reporters under the control of a calcineurin-dependent response element (CDRE). Using the reporters, we show that the rapid dynamics of calcineurin activation and deactivation can be followed by flow cytometry and fluorescence microscopy. This system is compatible with live/dead staining that excludes confounding dead cells from the analysis. The reporters provide technology to monitor calcineurin dynamics during stress and ageing and may serve as a drug-screening platform to identify novel antifungal compounds that selectively target calcineurin.

## INTRODUCTION

The Ca^2+^/calmodulin-dependent serine/threonine phosphatase calcineurin (CN) is a central component of Ca^2+^ signaling across species. CN regulates diverse fundamental processes, ranging from cell wall synthesis, stress responses and adaptation in unicellular organisms [1–3] to proliferation, development, immune responses, apoptosis, synaptic plasticity and memory function in metazoans [4–7]. CN activity across various cell types and organisms requires a tight spatio-temporal regulation. Both excessive and insufficient activity of this phosphatase have been linked to human disease, in particular to diverse neurodegenerative disorders [8, 9]. Pharmacological calcineurin inhibitors like FK506 (tacrolismus) or cyclosporin A are in clinical use as immunosuppressants, in particular after organ transplantations [5]. As calcineurin signaling has been shown to contribute to the virulence of a variety of fungal pathogens, including species of *Aspergillus, Cryptococcus* and *Candida*, CN inhibitors – alone or in combination with established antifungal drugs – are also being evaluated for their therapeutic potential to combat fungal infections [10, 11].

Calcineurin consists of a regulatory (CnB) and a catalytic (CnA) subunit, which in *Saccharomyces cerevisiae* are encoded by *CNB1* and either *CNA1 or CNA2*, respectively [7]. CnB harbors four EF-hand motifs, which bind Ca^2+^ with different affinities and confer structural and functional properties [12, 13]. CnA comprises a CnB-binding domain, a calmodulin-binding domain and an autoinhibitory domain, which blocks the active site in resting conditions. Ca^2+^-dependent binding of calmodulin to the CnA/CnB-Ca^2+^ dimer activates the phosphatase complex by displacing the autoinhibitory domain [7]. Across phyla, CN targets share common recognition sites (PxIxIT and LxVP), which determine enzyme-substrate specificity [6]. In yeast, CN-induced changes in gene expression are mainly mediated by the transcription factor Crz1, the analogue of mammalian NFAT, which upon dephosphorylation by CN drives transcriptional reprogramming and adaptation [1, 3]. Apart from this transcriptional response, CN also dephosphorylates various other substrates, thereby directly impacting processes such as vesicle trafficking, Ca^2+^ homeostasis, and lipid metabolism [14].

Currently established systems to monitor cellular CN activity are based on variations of the calcineurin-dependent response element (CDRE) fused to a reporter gene [2, 14–16]. Dephosphorylation of Crz1 by CN and subsequent binding of Crz1 to CDREs drives reporter gene expression. The most commonly applied reporters are based on the bacterial *LacZ*, coding for β-galactosidase (β-gal) [1, 8, 12, 16, 17]. While the β-gal assay represents a basic and cost-effective approach, efficiently used to understand diverse aspects of gene expression [18], it comes with clear limitations: it requires a rather lengthy procedure that precludes efficient high-throughput screenings and does not allow for *in vivo* measurement of CN activity. Here, we present a reporter system based on GFP that enables rapid and large-scale determination of CN activity in unperturbed, living cells and allows for a simultaneous discrimination between live and dead cells via flow cytometry. In addition, we utilize a GFP variant fused to a destabilizing tag (degron) to assess CN activity pulses and magnitudes in higher temporal resolution and demonstrate how dead cell populations confound a proper read-out of CN activity.

## RESULTS AND DISCUSSION

### GFP and GFP^PEST^ reporters capture CN activation

**Figure 1:**
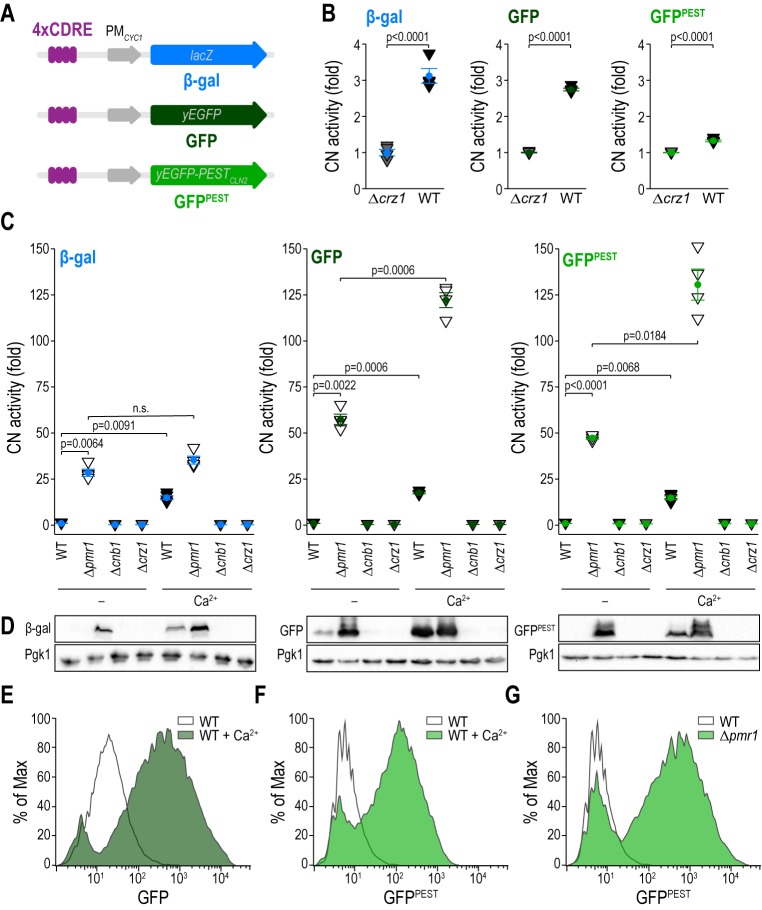
FIGURE 1: GFP and GFP^PEST^ function as reporters of calcineurin activity. **(A)** Schematics of pAMS366-4XCDRE-lacZ, pAMS366-4xCDRE-GFP and pAMS366-4xCDRE-GFP^PEST^ plasmids encoding reporters for CN activity. **(B)** CN activity was determined via β-gal activity or via flow cytometric quantification of GFP fluorescence intensities in exponentially growing wild type and Δ*crz1* cells equipped with either pAMS366-4xCDRE-lacZ, pAMS366-4xCDRE-GFP or pAMS366-4xCDRE-GFP^PEST^. Values are shown as fold of Δ*crz1* cells. Means ± SEM, n = 4. **(C)** CN activity was measured as in (B) in exponentially growing wild type, Δ*pmr1*, Δ*cnb1* and Δ*crz1* cells equipped with the indicated reporter plasmid. For stimulation of CN activity, cells were treated with 50 mM Ca^2+^ 1 h prior to measurement. Fold of untreated wild type cells is shown. Dead cells were excluded from the analysis via propidium iodide (PI) staining. Means ± SEM, n = 4. **(D)** Representative immunoblots of protein extracts from cells described in (C). Immunoblots were decorated with antibodies against β-gal or GFP, respectively, and Pgk1 as loading control. **(E-G)** Histograms of cells quantified in (C) indicating the shift in green fluorescence intensity of wild type cells with and without 50 mM Ca^2+^ treatment and Δ*pmr1* cells equipped with the GFP (E) or the GFP^PEST^ reporter (F, G).

To enable large-scale analysis of CN activity in unperturbed, living yeast cells, we generated reporter constructs coding for a yeast-optimized green fluorescent protein (yEGFP; hereafter referred to as GFP) under the control of a 4-fold repeat of CDRE, allowing Crz1-driven expression of GFP upon dephosphorylation of this CN-responsive transcription factor **(Fig. 1A)**. While the stability of GFP and thus its accumulation over time might be advantageous for instance in scenarios requiring high sensitivity due to marginal CN activation, it also limits its application as reporter molecule to analyze dynamic and transient changes in gene expression. Thus, we additionally employed GFP destabilized by fusion to a PEST-motif derived from the cyclin Cln2, marking it for rapid proteasomal degradation [23, 25]. We directly compared Crz1/CDRE-driven expression of β-gal, GFP and GFP^PEST^ to assess CN activity in (i) dividing, unstressed wild type cells, (ii) upon genetic disruption of cellular Ca^2+^ homeostasis, and (iii) upon administration of high external Ca^2+^. First, we monitored basal CN activity in resting conditions in wild type versus Δ*crz1* cells transformed with the different reporter plasmids. Here, Δ*crz1* cells served as background, and obtained β-gal activity values as well as GFP fluorescence intensities are depicted as fold of Δ*crz1* to allow comparison between the different reporter molecules **(Fig. 1B)**. Determination of β-gal activity in cell lysates as well as flow cytometric quantification of GFP fluorescence intensity in living cells revealed a ∼3-fold increase of CN activity compared to Δ*crz1* cells. A lower CN activity was detectable in cells expressing the short-lived GFP^PEST^ as reporter, consistent with PEST-driven destabilization and enhanced proteasomal degradation of this molecule **(Fig. 1B)**. To evaluate the different reporter molecules in a context of constitutively active CN, we used cells devoid of Pmr1, an ER/Golgi-localized Ca^2+^/Mn^2+^ ATPase that pumps Ca^2+^ from the cytosol into lumenal stores. Cells lacking Pmr1 are known to have elevated cytosolic Ca^2+^ levels and increased CN activity in resting, uninduced conditions [26, 27]. Flow cytometric quantification of fluorescence intensities *in vivo* in comparison with measurement of β-gal activity in lysates demonstrated that both GFP and GFP^PEST^ efficiently captured the prominent increase in CN activity in cells devoid of Pmr1 **(Fig. 1C)**. Cells lacking the regulatory subunit of calcineurin (Δ*cnb1)* as well as Δ*crz1* cells served as negative controls. To allow direct comparison of the magnitude of CN activity captured by the three reporter molecules, relative change to wild type was plotted **(Fig. 1C)**. Using immunoblotting as an alternative read-out to flow cytometry, we found that similar to β-gal, the protein levels of GFP and GFP^PEST^ were markedly increased in absence of Pmr1 **(Fig. 1D)**. Next, we monitored the response to administration of 50 mM Ca^2+^, a regime known to activate CN signaling and nuclear translocation of Crz1 [15]. Quantification of β-gal activity and fluorescence intensities revealed a 10-fold increase of CN activity in wild type cells 1 h after Ca^2+^ administration for all three reporter molecules. Moreover, both GFP as well as GFP^PEST^ intensities in cells with constitutively high CN activity (Δ*pmr1)* further increased upon additional Ca^2+^ treatment, effectively capturing CN activities of high amplitudes **(Fig. 1C)**. Corresponding histograms of GFP **(Fig. 1E)** and GFP^PEST^ **(Fig. 1F, G)** intensities of untreated versus Ca^2+^-treated wild type cells as well as of wild type cells versus Δ*pmr1* cells depict the shift in fluorescence intensities as a read-out for CN activity. In sum, these fluorescence-based systems function as reporters of CN activity during genetic (permanent) and pharmacological (transiently induced) activation of CN and are able to effectively capture CN activities of different magnitudes.

### Reporter protein stability assessed via immunoblotting

**Figure 2:**
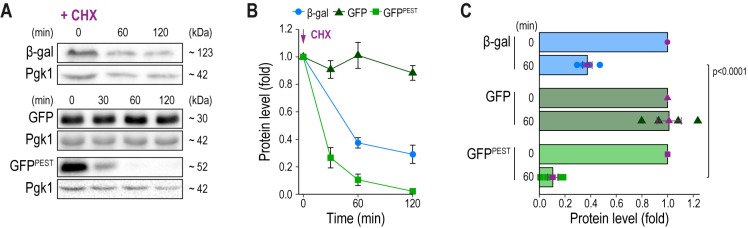
FIGURE 2: Reporter protein stability assessed via immunoblotting. **(A-C)** Representative immunoblots (A) and corresponding densitometric quantification (B, C) of reporter protein levels in exponentially growing cells lacking Pmr1 equipped with either pAMS366-4xCDRE-lacZ, pAMS366-4xCDRE-GFP or pAMS366-4xCDRE-GFP^PEST^ at indicated times points after addition of cycloheximide (CHX) to a final concentration of 100 mg/L. Blots were decorated with antibodies against β-galactosidase or GFP, respectively, and Pgk1 as loading control. Molecular weight (kDa) as estimated by Page Ruler Protein standard. Values represent fold to t = 0 min. Means ± SEM, n = 4.

Slow turnover of any reporter molecule obstructs the accurate detection of rapid downregulation of gene expression. We monitored protein stability of β-gal, GFP and GFP^PEST^ after inhibition of *de novo* protein synthesis with cycloheximide in cells with permanently active CN due to the absence of Pmr1 **(Fig. 2A–C)**. As expected, immunoblotting demonstrated that GFP was highly stable, and no decay was detectable within 120 min after cycloheximide treatment. While β-gal protein levels dropped to about 40% after 60 min, no further degradation was observed over the analyzed time frame, limiting its usefulness as a reporter to detect rapid downregulation of gene expression. In contrast, arrest of translation resulted in a fast reduction of GFP^PEST^ protein levels. 60 min after cycloheximide addition, about 90% of GFP^PEST^ was degraded by the proteasome, and after 120 min, GFP^PEST^ was completely turned over **(Fig. 2A–C)**. Thus, the proteasomal turnover of GFP^PEST^ is well-suited to capture rapid downregulation of CN activity.

### GFP^PEST^ monitors transient changes in CN activity

**Figure 3:**
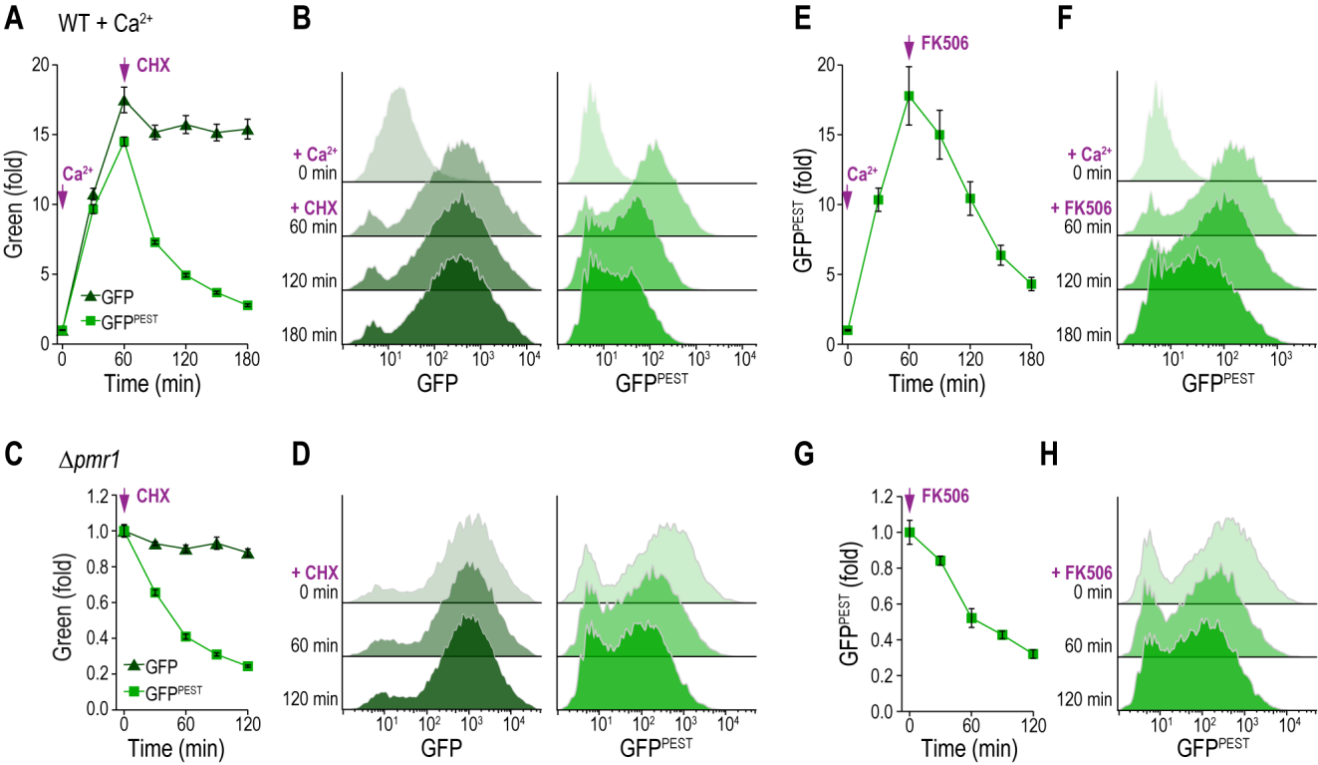
FIGURE 3: GFP^PEST^ as a reporter for transient changes in CN activity. **(A-D)** Flow cytometric quantification of GFP intensities (A, C) and corresponding histograms of selected time points (B, D) after addition of cycloheximide (CHX) to wild type cells pre-treated with 50 mM Ca^2+^ for 1 h (A, B) or to Δ*pmr1* cells (C, D) equipped with either pAMS366-4xCDRE-GFP or pAMS366-4xCDRE-GFP^PEST^. **(E-H)** Flow cytometric quantification of GFP intensities (E, G) and corresponding histograms of selected time points (F, H) after addition of 0.5 µM FK506 to wild type cells pre-treated with 50 mM Ca^2+^ for 1 h (E, F) or to Δ*pmr1* cells (G, H) equipped with pAMS366-4xCDRE-GFP^PEST^. Dead cells were excluded from the analysis via propidium iodide (PI) staining. Means ± SEM, n = 4.

Next, we tested whether the protein synthesis arrest of stable GFP and destabilized GFP^PEST^ was also readily detectable using fluorescence as a read-out. Therefore, we quantitatively evaluated the fluorescence intensities of the CN activity reporter molecules via flow cytometry. We treated wild type cells with 50 mM Ca^2+^ to stimulate CN activity, which triggered a rapid increase in both GFP and GFP^PEST^ intensities **(Fig. 3A, B)**. Consistent with the protein expression levels, subsequent cycloheximide addition led to a fast decline of GFP^PEST^ but not GFP fluorescence. Corresponding histograms show that both GFP and GFP^PEST^ faithfully reported on the CN activation upon Ca^2+^ addition, while only GFP^PEST^ captured the shut down upon cycloheximide treatment **(Fig. 3A, B)**. In this line, stop of translation in Δ*pmr1* cells, which exhibit permanently high CN activity, triggered a rapid decline of GFP^PEST^ fluorescence, while GFP intensities were mainly unaffected **(Fig. 3C, D)**. Ultimately, we confirmed that GFP^PEST^ captures also a decline of CN activity upon pharmacological inhibition of CN. To this end, we used FK506, which in complex with the immunophilin FKBP12 specifically inhibits CN across species [7, 11]. Indeed, addition of FK506 to Ca^2+^-treated wild type cells **(Fig. 3E, F)** as well as to Δ*pmr1* cells with high resting CN activity **(Fig. 3G, H)** led to a rapid drop in CN activity as assessed by flow cytometric quantification of GFP^PEST^ intensities. In sum, both reporters permit *in vivo* monitoring of CN activity and recapitulate the rapid stimulation of this phosphatase. While GFP^PEST^ additionally enables the assessment of CN deactivation due to its fast proteasomal turnover, the accumulation of the more stable GFP can serve to monitor marginal CN activities close to background.

### Flow cytometric analysis enables simultaneous exclusion of dead cells

**Figure 4:**
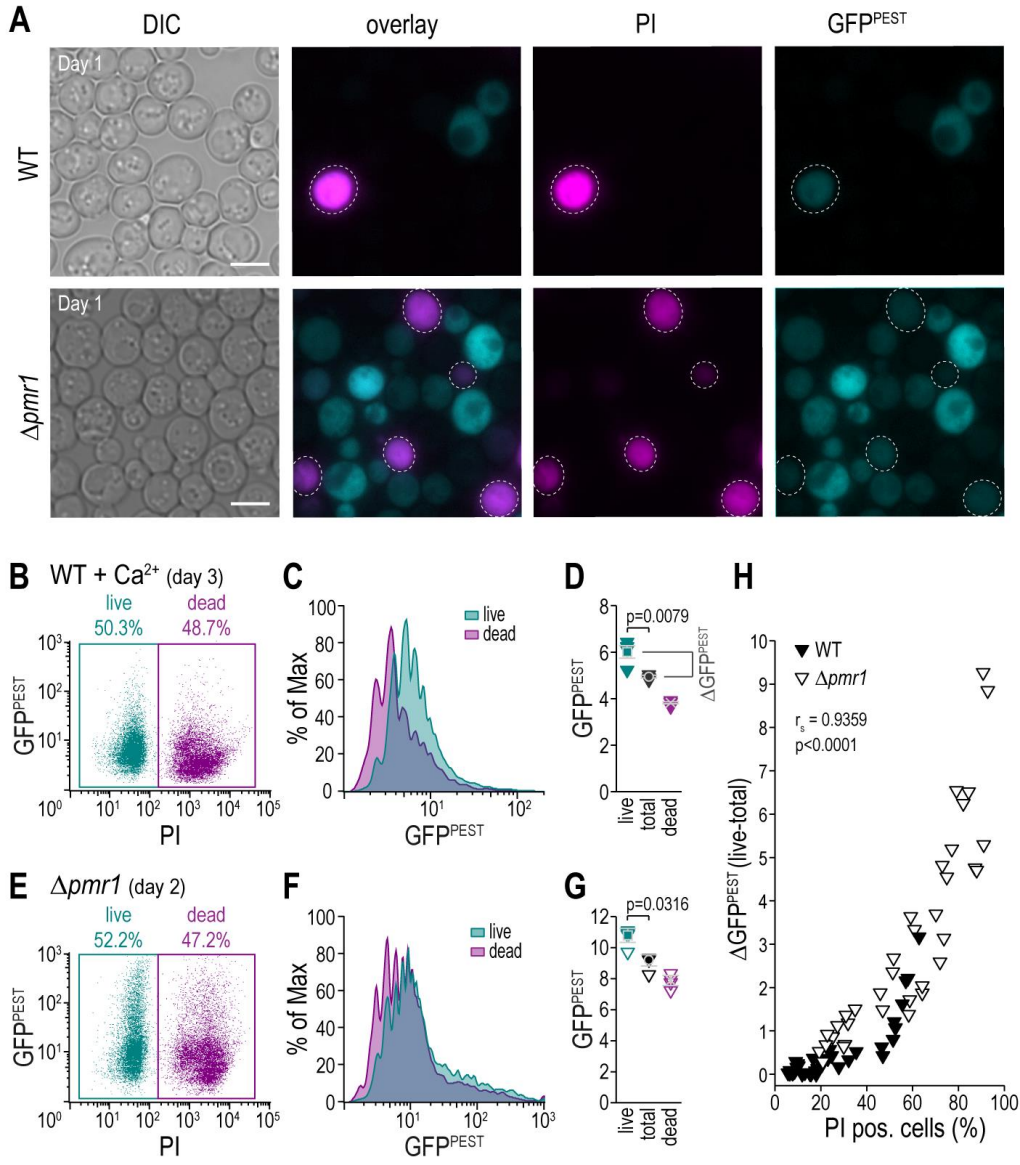
FIGURE 4. A fluorescent reporter for CN activity allows for simultaneous exclusion of dead cells via flow cytometry. **(A)** Representative micrographs of wild type and Δ*pmr1* cells equipped with pAMS366-4xCDRE-GFP^PEST^ and stained with propidium iodide (PI) to indicate loss of plasma membrane integrity and thus cell death. Scale bar = 5 µm. **(B-G)** Flow cytometric analysis of PI-stained aged wild type cells treated with Ca^2+^ for 1 h as well as Δ*pmr1* cells equipped with pAMS366-4xCDRE-GFP^PEST^. Respective dot plots (B, E), histograms of GFP^PEST^ intensities of PI negative (live) and PI positive (dead) cells (C, F) and mean GFP^PEST^ intensities of total, live and dead cell populations (D, G) are shown. **(H)** Simultaneous quantification of cell death via PI staining and CN activity in cells equipped with pAMS366-4xCDRE-GFP^PEST^. Wild type and Δ*pmr1* cells of different age and upon treatment with different concentrations of Ca^2+^ were analyzed, and the difference of GFP^PEST^ fluorescence intensity between total and live cell populations (ΔGFP^PEST^ (live-total)) was plotted against the percentage of PI positive cells.

Dead or dying cells in a culture confound the result of any readout if not accounted for, especially as those cells and debris thereof contribute to the optical density that frequently is used for normalization. The use of fluorescent reporters combined with a live/dead staining allows for the simultaneous analysis of CN activity and cell death. We employed propidium iodide (PI), indicative of loss of plasma membrane integrity, to discriminate between live and dead cell populations **(Fig.4A–G)**. As expected, flow cytometric evaluation and appropriate gating of the distinct subpopulations demonstrated that simply assessing CN activity in the total cell population leads to a significant underestimation of the actual CN activity of living cells **(Fig. 4B–G)**. To evaluate the impact of dead cell populations on actual CN activity, we simultaneously monitored cell death and CN activity in wild type and Δ*pmr1* cultures of different cellular age and upon treatment with various concentrations of Ca^2+^. For each condition, we determined the shift in GFP fluorescence intensity upon exclusion of the dead cell population (ΔGFP^PEST^) **(Fig. 4H)**. Plotting ΔGFP^PEST^ against the percentage of cell death determined via PI staining shows a clear positive correlation (Spearman correlation coefficient r_s_ = 0.9359, p<0.001, **Fig. 4H)**. Thus, combining a fluorescent reporter molecule with a live/dead staining facilitates accurate assessment of CN activity in cultures with larger populations of dead cells, for instance upon ageing, genetic modification or drug treatment. In addition, a flow cytometric read-out of fluorescence will also enable a direct correlation between the degree of CN activity in each cell and any other parameter using appropriate fluorescent dyes or tags.

Collectively, our results demonstrate that CDRE-driven expression of GFP and GFP^PEST^ serves as effective reporter to monitor CN activity via flow cytometry, fluorescence microscopy or immunoblotting. Use of the short-lived GFP^PEST^ enables the assessment of rapid and dynamic changes in CN activity, whereas the stability of GFP render it suitable to report on more subtle changes in base-line activities of the phosphatase in unstressed, resting cells. In combination with a live/dead staining, our system represents an efficient screening platform to simultaneously evaluate compounds for CN inhibitory activity and toxicity.

## MATERIALS AND METHODS

### Yeast strains and culture conditions

*S. cerevisiae* strains used in this study were all derived from BY4741 (*MAT*a; *his3*Δ1; *leu2*Δ0, *met15*Δ0; *ura3*Δ0, Euroscarf). *CRZ1* (BY4741 *crz1*::hphNT1) and *CNB1* (BY4741 *cnb1*::hphNT1) deletion mutants have been described previously [19]. The *PMR1* deletion mutant was generated via homologous recombination following established protocols [20]. Oligonucleotides 5'-CAG CAC AGA CGT AAG CTT AAG TGT AAG TAA AAG ATA AGA TAA TCA GCT GAA GCT TCG TAC GC-3' and 5'-TAA CAG AGA CAG TCC AAC GGC GTA GTT GAA CAT TTT GTT GCA TAG GCC ACT AGT GGA TCT G-3' used to amplify the gene disruption cassette from pUG6 were designed to leave *HUR1* intact, which overlaps with the C-terminal region of *PMR1* on the complementary strand (BY4741 *pmr1*::kanMX4 *HUR1* intact). Plasmid transformation was performed as previously described [21]. Cells were grown in synthetic complete medium containing 0.17% yeast nitrogen base (Difco, BD Biosciences), 0.5% (NH_4_)_2_SO_4_, 2% glucose, 30 mg/l adenine and 30 mg/l of all amino acids (except for 80 mg/l histidine and 200 mg/l leucine) and lacking uracil for reporter plasmid selection. All components were prepared separately as stocks and mixed after autoclaving (20 min, 121°C, 210 kPa): 10x YNB with (NH_4_)_2_SO_4_, 10x amino acids + adenine mixture, 20% glucose. Cells were treated with 50 mM CaCl_2_ (stock 2 mM CaCl_2_), 100 mg/L cycloheximide (stock 10 mg/ml; Sigma) at indicated time points. Overnight cultures incubated for 16-20 h where used to inoculate cultures to OD 0.1. For standard experiments (cell death analysis, fluorescence intensity measurements and epifluorescence microscopy) cells were cultivated in 96-deep well plates at 28°C, 999 rpm. For experiments requiring larger amounts of cells (β-gal assay and immunoblot analysis), cells were cultivated in 125 ml baffled Erlenmeyer flasks with cellulose stoppers at 28°C, 145 rpm. To rule out clonogenic variation, all experiments were performed with four different transformants.

### Construction of fluorescence-based reporter plasmids

The reporter constructs generated and used in this study are illustrated in Figure 1A and have been deposited at Addgene (IDs: 138657 and 138658), including plasmid maps and sequencing data. **Table 1** summarizes the most relevant features of the constructs. For the GFP version (pAMS366-4xCDRE-GFP), yEGFP was amplified from pYM25 [22] with primers 5'-ATC TGG ATC CAT GTC TAA AGG TGA AGA ATT ATT CAC-3' and 5'-ATC TGA GCT CTT ATT TGT ACA ATT CAT CCA TAC C-3' and ligated into pAMS366-4xCDRE (kind gift from M. Cyert) [1], cut with restriction enzymes BamHI and SacI (Thermo Scientific FastDigest). For the GFP^PEST^ version (pAMS366-4xCDRE-GFP^PEST^), pAMS366-4xCDRE was cut with BamHI (Thermo Scientific FastDigest) and ligated with yEGFP3-PEST_CLN2_ amplified from pSVA13 (kind gift from S. Avery) [23] with primers 5'-ATC TGG ATC CAT GTC TAA AGG TGA AGA ATT ATT CAC-3' and 5'-ATC TGG ATC CCT ATA TTA CTT GGG TAT TGC CCA TAC C-3'. Proper insertion of the PCR product into the vector backbone was confirmed by sequencing (Eurofins) with primers 5'-GTG GGT TTA GAT GAC AAG GGA GAC G-3' and 5'-CGT ACT GTG AGC CAG AGT TG-3' (for the GFP variant) and primers 5'-GTG GGT TTA GAT GAC AAG GGA GAC G-3', 5'-CAC AAA TTT TCT GTC TCC G-3' and 5'-GTC TTG TTA CCA GAC AAC C-3' (for the GFP^PEST^ variant).

**TABLE 1.** Specifications for pAMS366-4xCDRE-GFP and pAMS366-4xCDRE-GFP^PEST^.

	4xCDRE-GFP	4xCDRE-GFP^PEST^
Vector backbone	pAMS366 [1]	pAMS366 [1]
Copy number	high (2 µ)	high (2 µ)
Reporter	yeGFP [22]	yEGFP3-PEST_CLN2_ [23]
Reporter half-life	> 120 min	< 30 min
Auxotrophic marker	URA3	URA3
Antibiotic resistance	Amp^R^	Amp^R^

### β-gal assay

β-gal assay was essentially performed as described in [24]. In brief, 1 OD of cells was harvested, washed and resuspended in 250 µl Z-buffer (60 mM Na_2_HPO_4_*7 H_2_O, 40 mM Na_2_H_2_PO_4_*H_2_O, 10 mM KCl, 1 mM MgSO_4_, adjusted to pH 7.0 and 40 mM 2-mercaptoethanol). An aliquot of this suspension was used to measure OD_600_ for normalization. The remaining cells were lysed by addition of 0.01% SDS and 10% chloroform (final concentrations) and vortexing. To start and stop the enzymatic reaction, 0.36 mg/ml ONPG in Z-buffer and 0.2 M Na_2_CO_3_ (final concentrations) were added, respectively. After centrifugation (400 g, 5 min, 21°C), an aliquot of the suspension was taken to measure OD_405_ at a 2300 EnSpire Multimode Plate Reader (PerkinElmer) equipped with an excitation double monochromator (application: well area scan, averaging 6 measurement repeats). β-gal activity was calculated as (1000 x OD_405_ x dilution factor)/(OD_600_ x reaction time x volume in reaction). Per genotype and condition, four isogenic mutants were analyzed.

### Immunoblot analysis

6 OD of cells were harvested and lysed with 250 µl buffer containing 1.85 M NaOH and 7.5% β-mercaptoethanol. The suspension was incubated at 4°C for 10 min, 250 µl 55% trichloroacetic acid were added and incubated again at 4°C for 10 min. After centrifugation (4°C, 10 min, 10 000 g) trichloroacetic acid was removed and pellets were resuspended in Urea Loading Buffer (200 mM Tris/HCl, 8 M urea, 5% SDS, 1 mM EDTA, 0.02% bromophenol blue, 15 mM DTT, pH 6.8). SDS-PAGE with Tris-glycine running buffer (250 mM Tris base, 0.2 M glycine, 0.05% SDS (w/v)) was performed in a SE250 Mighty Small II Mini Vertical Protein Electrophoresis Unit (Hoefer) according to manufacturer's settings. For wet protein transfer onto a PVDF membrane (T830.1, Roth) with CAPS buffer (10 mM CAPS, 10% EtOH, pH 11) a TE22 Mighty Small Transfer Tank (Hoefer) was used. Blocking was performed with 5% milk powder in Tris buffered saline (50 mM Tris, 150 mM NaCl, pH 7.4). For immunoblotting anti-GFP (1:2500, 11814460001, Roche), anti-Pgk1 (1:10000, 459250, Invitrogen) and a peroxidase-conjugated secondary anti-mouse antibody (1:10000, A9044, Sigma-Aldrich) were used. Chemiluminescence imaging was performed on a ChemiDoc^TM^ XRS+ (BioRad) with ImageLab software (v 5.2.1) and the application Chemi Hi Resolution (no illumination, no filter, 2x2 binning, manual exposure times). The same software was used for densitometric quantification.

### Cell death analysis, epifluorescence microscopy and flow cytometry

PI staining as an indicator of loss of membrane integrity was used to assess cell death and was essentially performed as previously described [19]. In brief, approximately 1x10^6^ cells per sample were harvested in 96-well plates and incubated for 5 min with PI (81845, Sigma), final concentration 500 ng/ml in phosphate buffered saline (25 mM potassium phosphate, 0.9% NaCl; adjusted to pH 7.2). Micrographs were recorded using a ZEISS Axioplan2 microscope, (63x/1.40 oil objective, Cy5 (propidium iodide) and FITC (GFP) filters) with ZEISS Axio cam MRm camera and AxioVision40x64 software (v4.9.1.0). Flow cytometry was performed on a Guava easyCyte 5HT equipped with a 50 mW 488 nm laser (blue) and the following filters: 488/16 (SSC), 525/30 (green), 695/50 (red) (Merck group). Per genotype and condition, four isogenic mutants were analyzed. All samples were measured in the staining solution and each sample was resuspended automatically by mixing for 5 seconds before acquisition. Per sample, 5000 events were recorded (threshold parameter FSC-HLog, value 100). Data was acquired and analyzed with InCyte software (3.1).

### Data preparation and statistical analysis

Data were analyzed and graphs were generated with R version 3.5.1 (base, ggplot2 and dplyr packages) or FlowJo 10.6.1 (histograms and dot plots Fig. 4B, E). Figures were prepared in Adobe Illustrator CC 2017. Data are presented as dot plots or line graphs and, where applicable, with means and error bars showing standard error of mean (SEM). Histograms contain concatenated data of all transformants analyzed. Y-axis in all histograms is % of Max, where the mode in every population is scaled to 100%. For all data sets, normal distribution (Shapiro-Wilk's) and homogeneity of variances (Levene's) was confirmed (OriginPro 2017 b9.2.4.380) (except data depicted in Fig. 4H, please see below). To compare reporter protein levels (between-subject) over time (within-subject), a two-way ANOVA mixed design was performed with Tukey post hoc test for one variable (protein levels, Fig. 2C). For comparisons between two groups, an unpaired two-way Student's t-test was performed (Fig. 1B). For comparison of fold changes in Figure 1C, all conditions were treated as individual groups (independent variable) and analyzed with Brown-Foresythe and Welch ANOVA with Dunnett's T3 multiple comparison (Prism GraphPad v8.2.1) as there was no homogeneity of variances. Normality was given for all groups except for untreated Δ*cnb1* cells equipped with GFP^PEST^, which was ignored due to equal sample size in all groups. Cell death and ΔGFP^PEST^ were correlated with two-tailed Spearman rank test (nonparametric, as data was not normally distributed, Fig. 4H). P values are stated with 4 decimals or as n.s. (not significant, p>0.05).

## Abbreviations:

β-gal: – β-galactosidase,
CDRE: – calcineurin-dependent response element,
CN: – calcineurin,
CnA: – catalytic subunit of CN,
CnB: – regulatory subunit of CN,
GFP: – green fluorescent protein,
PI: – propidium iodide.
